# Current levels of microplastic pollution impact wild seabird gut microbiomes

**DOI:** 10.1038/s41559-023-02013-z

**Published:** 2023-03-27

**Authors:** Gloria Fackelmann, Christopher K. Pham, Yasmina Rodríguez, Mark L. Mallory, Jennifer F. Provencher, Julia E. Baak, Simone Sommer

**Affiliations:** 1 Institute of Evolutionary Ecology and Conservation Genomics, Ulm University, Ulm, Germany; 2 Institute of Marine Sciences - Okeanos, University of the Azores, Horta, Portugal; 3 Biology, Acadia University, Wolfville, Nova Scotia Canada; 4 Ecotoxicology and Wildlife Health Division, Environment and Climate Change Canada, Ottawa, Ontario Canada; 5 Department of Natural Resource Sciences, McGill University, Sainte-Anne-de-Bellevue, Quebec Canada

**Keywords:** Microbiome, Environmental impact, Microbial ecology, Marine biology

## Abstract

Microplastics contaminate environments worldwide and are ingested by numerous species, whose health is affected in multiple ways. A key dimension of health that may be affected is the gut microbiome, but these effects are relatively unexplored. Here, we investigated if microplastics are associated with changes in proventricular and cloacal microbiomes in two seabird species that chronically ingest microplastics: northern fulmars and Cory’s shearwaters. The amount of microplastics in the gut was significantly correlated with gut microbial diversity and composition: microplastics were associated with decreases in commensal microbiota and increases in (zoonotic) pathogens and antibiotic-resistant and plastic-degrading microbes. These results illustrate that environmentally relevant microplastic concentrations and mixtures are associated with changes in gut microbiomes in wild seabirds.

## Abstract (web summary)

Consuming microplastics is known to harm marine wildlife in several ways, but effects on the microbiome are understudied. Here the authors demonstrate that two species of wild seabirds with larger amounts of microplastic in their guts had fewer commensal gut microbial species but more pathogens.

## Main

Microplastics represent an emerging threat to wildlife and human health^1,2^. These small (<5 mm) plastic particles contaminate bodies of water, soils and the air^1,3^. The omnipresence of microplastics has fostered broad research aimed at determining potential negative health effects on exposed animals, including humans^1,3^. Research has demonstrated that microplastics can deleteriously affect animals and their health^3^. Despite this work, our understanding of the effects of microplastic ingestion on gut microbiome communities is poor.

The microbiome is the collection of microbes in a given area of the body that has formed an evolutionary symbiotic relationship with its host species^4^. Thus, microbiomes are essential to host nutrition, physiology, immune function, development and even behaviour, and many diseases have been associated with altered gut microbiomes^5^. Microbiomes can change in taxonomic and functional diversity in animals subjected to anthropogenic stressors such as environmental pollution^6,7^. Along this line, laboratory studies revealed that microplastics may cause changes in gut microbiomes with negative health implications^8–10^. As a field in its infancy, however, effects of microplastics in wild populations are still unknown. Considering that levels of microplastic pollution are expected to rise and accumulate with time^11^, it is imperative to understand how wildlife health, reflected by the gut microbiome, is impacted.

**Extended Data Fig. 1:**
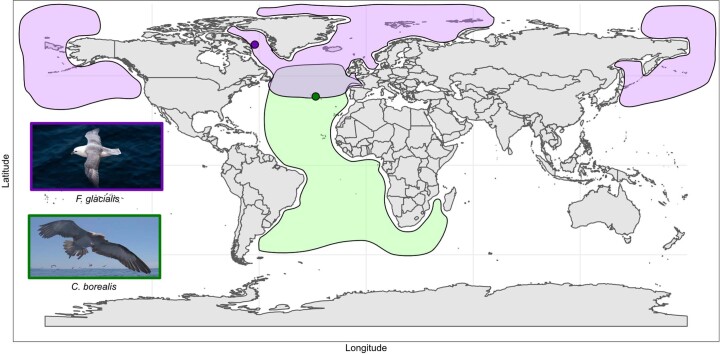
Sampling location and distribution of the study species *C. borealis* and *F. glacialis*. Cory’s shearwaters were collected at the edge of the North Atlantic subtropical gyre on the Azores archipelago (Portugal; dark green dot; distribution shown in green) and northern fulmars were collected near Qikiqtarjuaq, Nunavut in the Northwest Atlantic (dark purple dot; distribution shown in purple).

**Extended Data Fig. 2:**
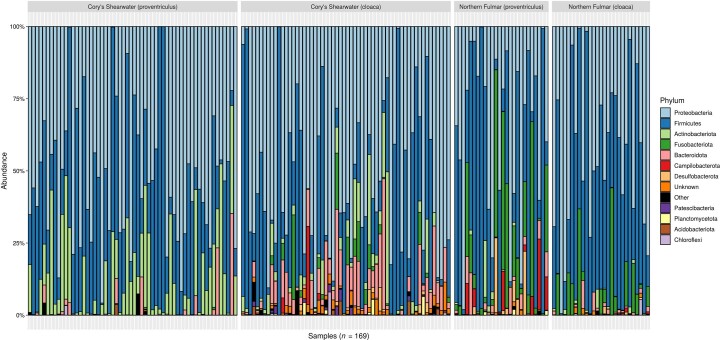
Microbiota composition at the phylum rank for the proventricular and cloacal microbiomes of Cory’s shearwaters and northern fulmars. The phyla within each sample (*n* = 169 samples obtained from 85 individual seabirds) are plotted by their relative abundance on the y-axis. Low-abundance phyla (prevalence < 0.1 and abundance < 10) are grouped together and labelled as “Other”.

In this Article, we studied the gut microbial response to varying degrees of microplastic ingestion, quantified by counting and weighing microplastics, in two different seabird species: Cory’s shearwaters (*Calonectris borealis*), *n* = 58 individuals, collected on the Azores archipelago in Portugal and northern fulmars (*Fulmarus glacialis*), *n* = 27 individuals, collected in Baffin Bay, Canada. Their distributions span both hemispheres (Extended Data Fig. 1). Both species ingest plastic debris, and in particular the fulmar is established as a plastics bioindicator^12–15^. By extending the focus from solely the gut microbiome (which in birds is usually determined by sampling the cloaca) to also include the microbiome of the proventriculus, we further aimed to determine if microplastic ingestion carries similar consequences on the microbiomes of the gastrointestinal tract (GIT) as it progresses along the digestive tract. Using 16S ribosomal RNA gene sequencing, we found that the most abundant phyla across the dataset were Proteobacteria (49.9%), Firmicutes (33.1%), Actinobacteriota (6.2%), Fusobacteriota (4.2%) and Bacteroidota (3.7%; Extended Data Fig. 2), which accounted for over 97% of the 4,602,578 reads.

## Results and discussion

**Fig. 1:**
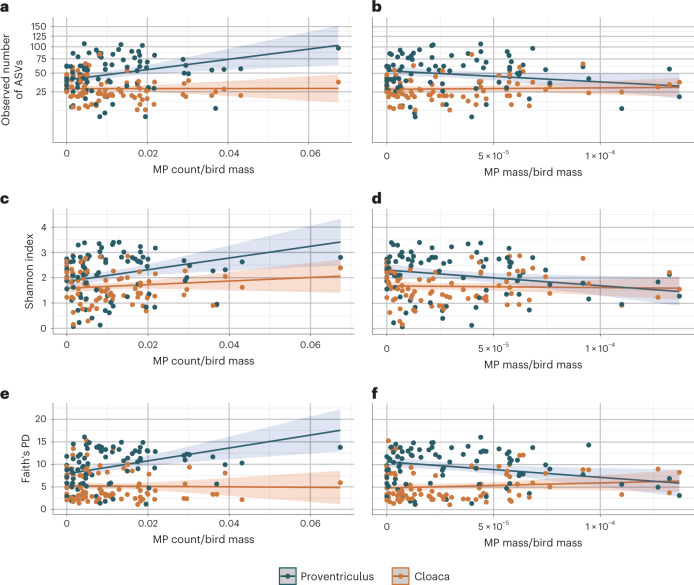
Correlations between microplastics (MP) and the alpha diversity of the proventricular and cloacal microbiomes in northern fulmar and Cory’s shearwater individuals. **a**–**f**, Each dot represents a microbiome sample that is coloured by the location within the GIT, either from the proventricular (blue dots, *n* = 85) or cloacal microbiome (orange dots, *n* = 84). Alpha diversity metrics: observed number of ASVs (note that the scale is non-linear due to the square root transformation of the alpha diversity values) (**a** and **b**), Shannon index (**c** and **d**) and Faith’s PD (**e** and **f**) are plotted in relation to the proportion of MP counts (MP count/individual bird mass; left) and the proportion of MP mass (MP mass/individual bird mass; right). The lines in each plot denote the predicted values based on the linear mixed model for that alpha diversity metric, and the shaded areas flanking the lines indicate the upper and lower 95% confidence intervals.

**Extended Data Fig. 3:**
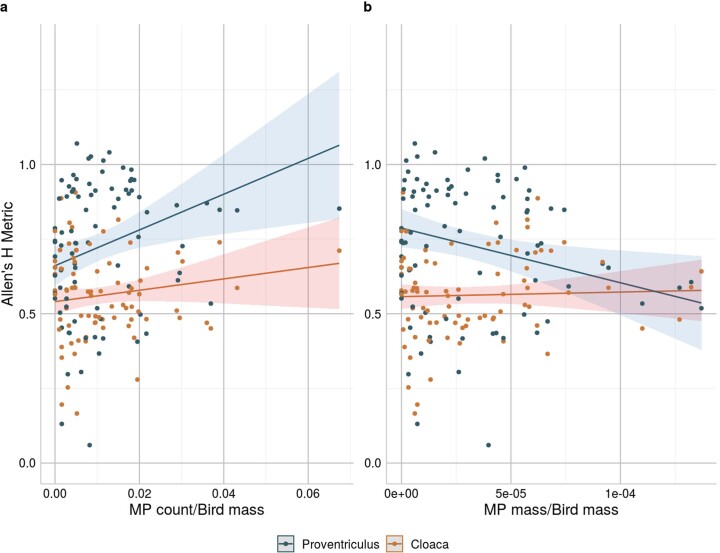
Correlations between microplastics (MP) and the alpha diversity of the proventricular and cloacal microbiomes in northern fulmar and Cory’s shearwater individuals. Each dot represents a microbiome sample that is colored by the location within the GIT, either from the proventricular (blue dots, *n* = 85) or cloacal microbiome (orange dots, *n* = 84). Alpha diversity metric Allen’s H metric is plotted in relation to **a** the proportion of MP counts (MP count/individual bird mass) and **b** the proportion of MP mass (MP mass/individual bird mass). The lines in each plot denote the predicted values based on the linear mixed model for the alpha diversity metric and the shaded areas flanking the lines indicate the upper and lower 95% confidence intervals.

Using linear mixed models and accounting for other biological and experimental variables (Supplementary Results), we tested if microbial alpha diversity (observed number of amplicon sequence variants (ASVs), Shannon index, Faith’s phylogenetic diversity (PD) and Allen’s H metric) of proventricular and cloacal microbiomes in the two species was associated with microplastics (counts and mass; Supplementary Results) and, by including interaction terms, if the effects of microplastics were similar between seabird species and throughout the GIT. For all alpha diversity metrics, microplastic count was significantly positively correlated with microbial alpha diversity in the proventriculus (observed number of ASVs: *β* = 0.67, *t*_81_ = 2.96, *P* = 0.004; Shannon index: *β* = 0.27, *t*_81_ = 2.85, *P* = 0.006; Faith’s PD: *β* = 1.68, *t*_81_ = 3.46, *P* < 0.001; Allen’s H metric: *β* = 0.07, *t*_81_ = 2.73, *P* = 0.007; Fig. 1, Extended Data Fig. 3 and Supplementary Table 1). These associations were significantly greater in the proventriculus than the cloaca (observed number of ASVs: *P* = 0.011; Faith’s PD: *P* = 0.001; with a trend for Shannon index: *P* = 0.084 and Allen’s H metric: *P* = 0.089), where this effect was close to zero (observed number of ASVs: *β* = 0.01; Shannon index: *β* = 0.08; Faith’s PD: *β* = −0.06; Allen’s H metric: *β* = 0.02).

In relation to mass of microplastics, birds with greater microplastic mass had significantly lower Shannon index (*β* = −0.20, *t*_81_ = −2.38, *P* = 0.020), Faith’s PD (*β* = −1.12, *t*_81_ = −2.47, *P* = 0.016) and Allen’s H metric (*β* = −0.06, *t*_81_ = −2.54, *P* = 0.013) and a trending negative correlation with the observed number of ASVs (*β* = −0.40, *t*_81_ = −1.95, *P* = 0.055) in the proventricular microbiome (Fig. 1 and Supplementary Table 1). These associations significantly differed between the proventricular and cloacal microbiome when considering Faith’s PD (*P* = 0.006) and Allen’s H metric (*P* = 0.020), and showed a trend for the observed number of ASVs (*P* = 0.073) and Shannon index (*P* = 0.090). In the cloaca, the association with cloacal Faith’s PD was—in contrast to the proventriculus—positive (*β* = 0.36), whereas it was close to zero for the observed number of ASVs (*β* = 0.05), Shannon index (*β* = −0.02) and Allen’s H metric (*β* = 0.01). Removing samples that could be considered outliers (less than the first percentile or greater than the 99th percentile) did not substantially change results (Supplementary Table 2). Similarly, models using microplastic count or mass without standardizing by bird mass also did not substantially change results (Supplementary Table 3).

In general, both microplastic count and mass were significantly correlated (positively and negatively, respectively) with alpha diversity, with greater correlations anteriorly (proventriculus) than posteriorly (cloaca), suggesting that effects of microplastics on microbial alpha diversity wane as they travel through the GIT. If microplastics act as vectors for pathogenic and/or foreign microbes^2,16^, then this mode of action could decrease along the GIT as hitchhiking microbes come into contact and compete with more resident microbes and have to survive stacking host immune defences^17^. Additionally, although the role of microplastics as vectors for hydrophobic organic chemicals remains unclear^18^, recent studies have shown their desorption in microplastics decreases exponentially over time in artificial gut solutions^19^ and are higher at greater temperatures and lower pH (ref. ^20^), which—at least in chickens—in lowest in the proventriculus^21^.

Further supporting the conclusion that microplastics could act as microbial vectors was the observation that Faith’s PD explained the highest amount of variation (Supplementary Results) and was most affected by microplastics. This suggests that microplastics can introduce not only a greater amount of microbes to GIT microbiomes (reflected by richness that also increased) but also a greater diversity of microbes from different evolutionary lineages. Notably, how the quantity of microplastics was measured revealed different potential impacts of microplastics on the GIT microbiome. While microplastic count was generally positively associated with alpha diversity, microplastic mass was generally negatively associated. Though microplastic count could increase alpha diversity by acting as vectors^2,16^, microplastic mass takes into account volume and density, the latter of which can be influenced by polymer type and properties^22^. Thus, mass probably reflects differences in polymer properties more than count. Since antimicrobial additives can be added to plastic polymers^22^, an increase in the mass of such a polymer could decrease microbial alpha diversity. Parsing out these differences would require more knowledge about the microplastic polymer types and their additives, and appears a fertile avenue for future research.

Model selection did not support the interaction between microplastics (count and mass) and host seabird species (Methods); thus, any correlations between microplastics and gut microbial alpha diversity were similar between northern fulmars and Cory’s shearwaters. This suggests that the effects observed in this study may apply widely in Procellariiformes that ingest microplastics.

**Extended Data Fig. 4:**
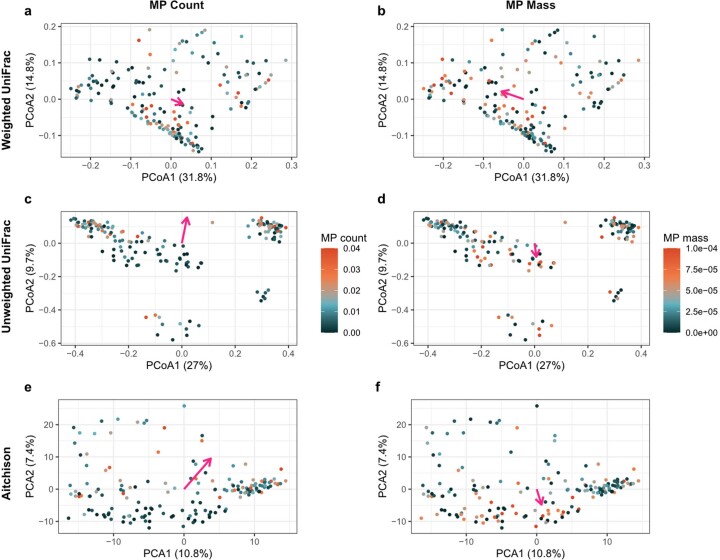
Ordination plots showing the correlations between microplastic (MP) count and mass and seabird GIT microbial beta diversity. Principle coordinate analysis (PCoA) ordination plots with **a**,**b** weighted UniFrac distances, **c**,**d** unweighted UniFrac distances, and **e**,**f** principle component analysis (PCA) ordination plots with Euclidean distances (Aitchison’s approach). Each dot represents a microbiome sample colored on a continuous scale by (**a**,**c**,**e**) the proportion of MP count (MP count/individual bird mass; *n* = 169) and (**b**,**d**,**f**) the proportion of MP mass (MP mass/individual bird mass; *n* = 169) and magenta arrows show the direction of the MP effects.

**Extended Data Fig. 5:**
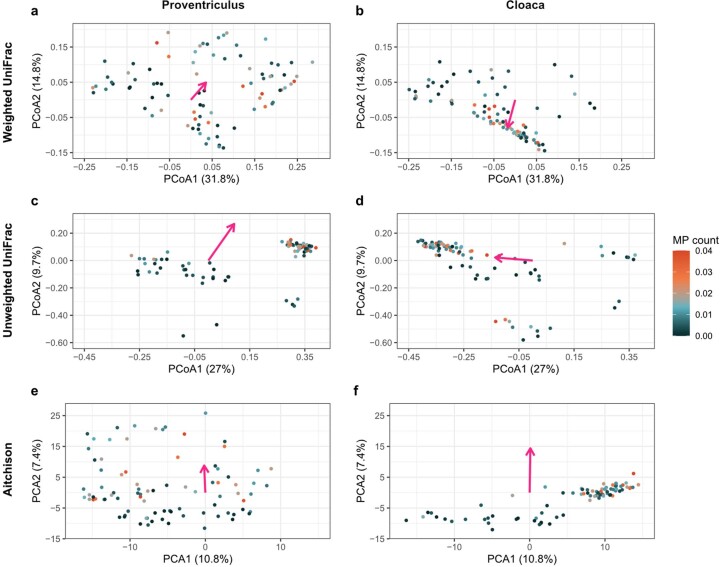
Ordination plots showing the correlations beween MP count and seabird proventricular versus cloacal microbial beta diversity. PCoA plots with **a**,**b** weighted UniFrac distances, **c**,**d** unweighted UniFrac distances, and **e**,**f** PCA plots with Euclidean distances (Aitchison’s approach) illustrate the effects of MP counts on seabird proventricular (**a**,**c**,**e**; *n* = 85) versus cloacal (**b**,**d**,**f**; *n* = 84) microbial beta diversity. Each dot represents a microbiome sample colored on a continuous scale by the proportion of MP count (MP count/individual bird mass) and magenta arrows show the direction of the MP effects.

**Extended Data Fig. 6:**
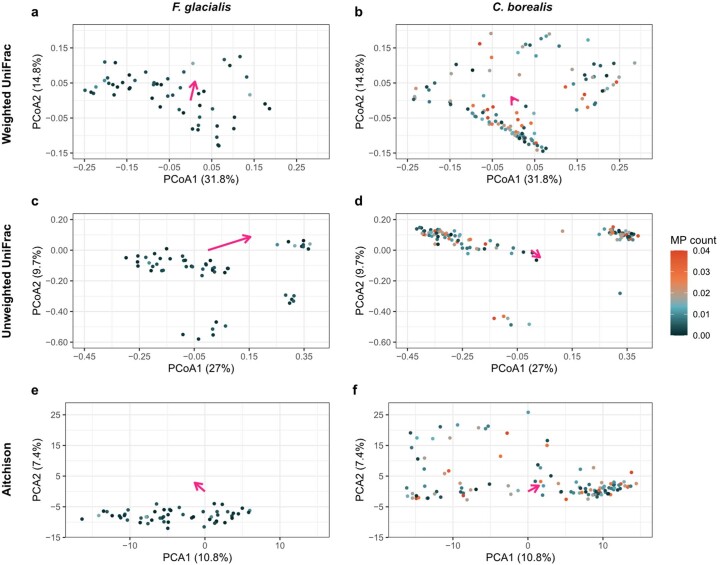
Ordination plots showing the correlations between MP count and northern fulmar versus Cory’s shearwater GIT microbial beta diversity. PCoA plots with **a**,**b** weighted UniFrac distances, **c**,**d** unweighted UniFrac distances, and **e**,**f** PCA plots with Euclidean distances (Aitchison’s approach) illustrate the effects of MP count on GIT microbial beta diversity in northern fulmars (**a**,**c**,**e**; *n* = 27) versus Cory’s shearwaters (**b**,**d**,**f**; *n* = 58). Each dot represents a microbiome sample colored on a continuous scale by the proportion of MP count (MP count/individual bird mass) and magenta arrows show the direction of the MP effects.

**Extended Data Fig. 7:**
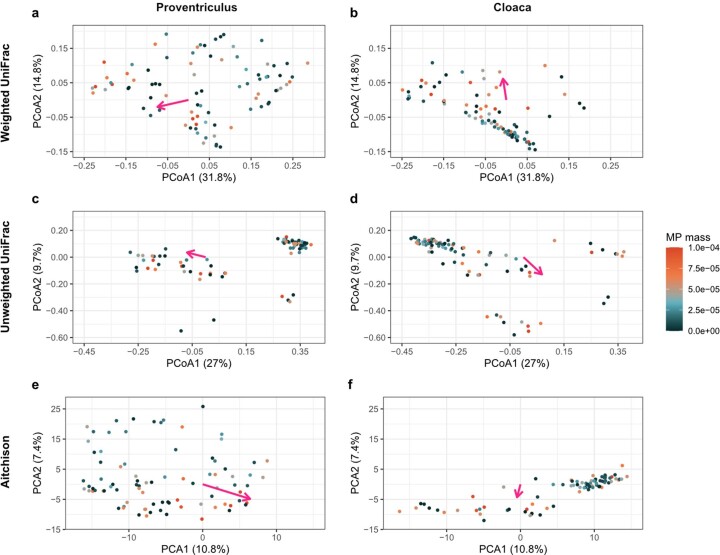
Ordination plots showing the correlations between MP mass and seabird proventricular versus cloacal microbial beta diversity. PCoA plots with **a**,**b** weighted UniFrac distances, **c**,**d** unweighted UniFrac distances, and **e**,**f** PCA plots with Euclidean distances (Aitchison’s approach) illustrate the effects of MP mass on seabird proventricular (**a**,**c**,**e**; *n* = 85) versus cloacal (**b**,**d**,**f**; *n* = 84) microbial beta diversity. Each dot represents a microbiome sample colored on a continuous scale by the proportion of MP mass (MP mass/individual bird mass) and magenta arrows show the direction of the MP effects.

**Extended Data Fig. 8:**
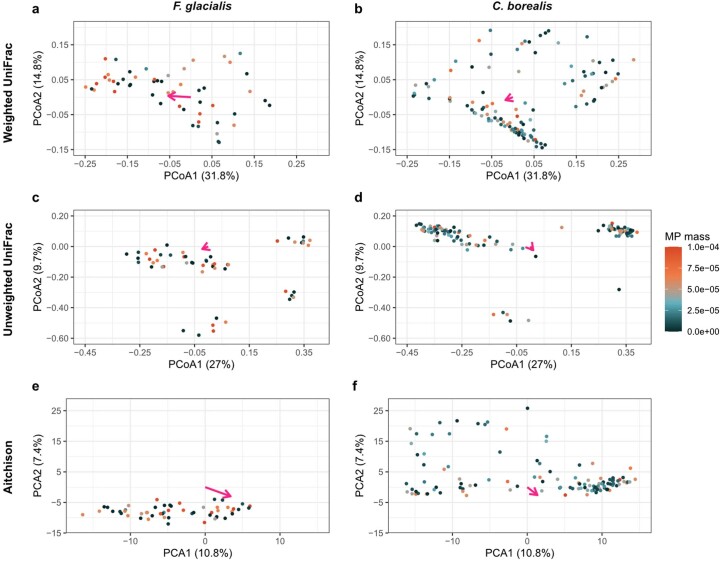
Ordination plots showing the correlations between MP mass and northern fulmar versus Cory’s shearwater GIT microbial beta diversity. PCoA plots with **a**,**b** weighted UniFrac distances, **c**,**d** unweighted UniFrac distances, and **e**,**f** PCA plots with Euclidean distances (Aitchison’s approach) illustrate the effects of MP mass on GIT microbial beta diversity in northern fulmars (**a**,**c**,**e**; *n* = 27) versus Cory’s shearwaters (**b**,**d**,**f**; *n* = 58). Each dot represents a microbiome sample colored on a continuous scale by the proportion of MP mass (MP mass /individual bird mass) and magenta arrows show the direction of the MP effects.

Next, we investigated if microplastics correlated with GIT microbial composition between individuals (beta diversity) and if these correlations were similar between seabird species and throughout the GIT. We did this using permutation tests implemented in vegan::adonis with 9,999 permutations (Supplementary Results)^23^. For visualization purposes, microplastics results were plotted in principal coordinates analysis (PCoA) plots (Extended Data Figs. 4–8), which are only able to represent two dimensions at a time, whereas these statistical results were evaluated across all the dimensions of the beta diversity matrices. When considering microplastic count, we found count to be significantly correlated with beta diversity when using weighted (*P* < 0.001) and unweighted (*P* < 0.001) UniFrac distances, as well as Euclidean distances within Aitchison’s log-ratio approach for compositional data (*P* < 0.001; Extended Data Fig. 4 and Supplementary Table 4). However, this depended not only on location of the microbiome within the GIT (weighted UniFrac: *P* = 0.008; unweighted UniFrac: *P* < 0.001; Aitchison: *p* = 0.001; Extended Data Fig. 5), but also on host seabird species (weighted UniFrac *p* = 0.043; unweighted UniFrac: *p* < 0.001; Aitchison: *P* < 0.001; Extended Data Fig. 6 and Supplementary Table 4). This means that microplastic count has different associations with beta diversity in the proventriculus versus cloaca, and between the species.

Moving from microplastic count to mass, we found mass to be significantly correlated with beta diversity when using unweighted UniFrac distances (*P* < 0.001) and Aitchison’s approach (*P* < 0.001) and a trend when using weighted UniFrac distances (*P* = 0.069; Extended Data Fig. 4). This depended on location of the microbiome within the GIT when considering weighted UniFrac distances (*P* = 0.016; Extended Data Fig. 7a,b) and Aitchison’s approach (*P* = 0.011; Extended Data Fig. 7e,f), as well as the host species being investigated (weighted UniFrac: *P* < 0.001; unweighted UniFrac: *P* < 0.001; Aitchison: *P* < 0.001; Extended Data Fig. 8 and Supplementary Table 4). Consequently, microplastic mass was associated with microbial composition of the proventriculus and cloaca differently only when considering weighted UniFrac distances and Aitchison’s approach, but not unweighted UniFrac distances. Moreover, this association depended on the host species in question. Results from models using microplastic count or mass without standardizing by bird mass showed similar results (Supplementary Table 5). Since the host species we investigated were collected in distant locations, belong to different genera and represented different age groups (adults versus fledglings), it was not surprising that their GIT microbiome compositions differed^24^, meaning the microbial taxa available to be shifted might not be the same. Future studies will be necessary to disentangle the roles of host phylogeny, age and geographic location in modulating effects of microplastics on wildlife gut microbiomes.

**Fig. 2:**
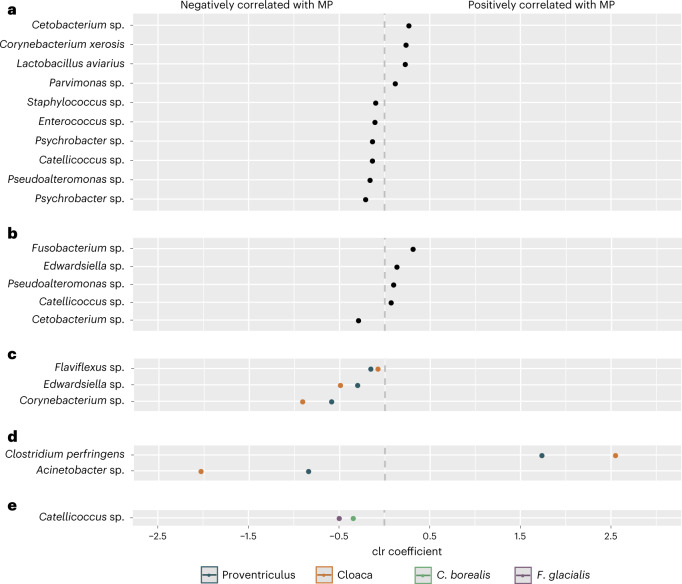
Differentially abundant ASVs associated with microplastics (counts and mass) identified by ANCOM. **a**–**e**, Each dot represents an ASV plotted by its taxonomic assignment on the *y* axis and in decreasing order of its centred log-ratio (clr) coefficient on the *x* axis. Thus, dots to the right of centre zero show a positive correlation with microplastics, whereas dots to the left show a negative correlation. Plotted ASVs were identified as differentially abundant by ANCOM (at *w*_0_ = 0.70) according to microplastic count (**a**), microplastic mass (**b**), the interaction between microplastic count and sample type (blue dots represent the proventriculus, red dots represent the cloaca) (**c**), the interaction between microplastic mass and sample type (blue dots represent the proventriculus, orange dots represent the cloaca) (**d**) and the interaction between microplastic counts and species (green dots represent Cory’s shearwaters, purple dots represent northern fulmars) (**e**). ANCOM identified 17 differentially abundant ASVs; however, 21 dots are shown here because 3 of the 17 ASVs are associated with microplastic counts (**a**) as well as microplastic mass (**b**; annotated as *Catellicoccus* sp., *Cetobacterium* sp. and *Pseudoalteromonas* sp.) and one ASV is associated with both microplastic mass (**b**) and the interaction between microplastic counts and sample type (**c**; annotated as *Edwardsiella* sp.).

To understand which taxa could be driving the associations between microplastics and microbial beta diversity, we performed an ANCOM test^25^, which determined 17 ASVs to be differentially abundant (Fig. 2 and Supplementary Results). Of these, ten were associated with microplastic count (Fig. 2a) and five were associated with microplastic mass (Fig. 2b). The genera *Catellicoccus*, *Cetobacterium* and *Pseudoalteromonas* were associated with both microplastic count and microplastic mass. Notably, as microplastic count increased, the abundance of resident microbiota associated with healthy hosts decreased, while the abundance of microbes known to be involved in disease, antibiotic resistance and plastic degradation and those considered to be zoonotic pathogens increased. For example, *Pseudoalteromonas*, which comprises marine bacteria usually associated with healthy organisms^26^, was negatively associated with microplastic count, as were known members of (sea)bird microbiota, such as *Psychrobacter*^26,27^, *Enterococcus*^28,29^, *Catellicoccus*^30,31^ and *Staphylococcus*^32,33^. In contrast, *Corynebacterium xerosis* was positively associated with microplastic count and has been identified as an emerging pathogen with potential to become zoonotic^34,35^, with its genus having shown plastic-degrading capabilities (database in ref. ^36^). Moreover, although *Lactobacillus aviarius* is a common member in avian microbiota^37^, an increased abundance is indicative of poor development in birds^29^. Positively correlated with microplastic count were *Parvimonas*, a predictor of colorectal cancer in humans^38^, and *Cetobacterium*, which is resistant to the antibiotic vancomycin^39^. *Clostridium perfringens*, which had the greatest positive association with microplastic mass and a larger association with the cloacal versus proventricular microbiome, is a pathogen in chickens that produces extracellular toxins that can cause avian necrotic enteritis as well as life-threatening gas gangrene and food poisoning in humans^40^. Other potential pathogens associated with microplastic mass were *Fusobacterium*^41^ and *Edwardsiella*^42,43^.

The genera *Cetobacterium* and *Fusobacterium* were also associated with plastic ingestion in captive loggerhead sea turtles (*Caretta caretta*) rescued from the Northwestern Adriatic Sea^44^. This suggests that microplastics may have similar impacts on gut microbial communities not only between closely related species such as the seabirds in our study, but also between more distantly related species that inhabit similar environments.

Of the 17 differentially abundant ASVs in total, 3 were negatively associated with the interaction between microplastic count and GIT location (Fig. 2c; *Flaviflexus*, *Edwardsiella* and *Corynebacterium*). Two ASVs were associated with the interaction between microplastic mass and GIT location: *Clostridium perfringens*, which was positively correlated, and *Acinetobacter*, which was negatively correlated (Fig. 2d). For both ASVs, the magnitude of the correlation was greater in the cloacal than the proventricular microbiome. ANCOM revealed one ASV belonging to the genus *Catellicoccus* to be negatively associated with the interaction between microplastic count and host seabird species, but no ASVs to be associated with the interaction between microplastic mass and host species (Fig. 2e).

We explored if the effects of microplastics were tied to host body condition using the scaled mass index calculated using individual tarsus length and body mass. However, the only significant predictors of body condition were host species (*β* = −0.76, t = -3.98, *p* =< 0.001) and the interaction between species and microplastic count (*β* = −0.94, *t* = −3.62, *P* ≤ 0.001), but not microplastic count alone (Supplementary Table 6). Thus, any effects that microplastics may have on host health were not captured by our measurement of body condition. Moreover, body condition was not a significant predictor in any alpha diversity models (Supplementary Table 7). In our beta diversity models, body condition significantly predicted unweighted UniFrac (*P* = 0.007) and Aitchison distances (*P* < 0.001; Supplementary Table 8). Therefore, although microplastics are linked with aspects of microbial composition, the mechanism by which microplastics could cause microbial changes does not seem to be linked to body condition.

To summarize, we found that ingested microplastics correlated with microbial diversity and composition throughout seabird GITs. Associations between microplastics and alpha diversity did not depend on host seabird species, whereas their correlations with beta diversity were host species specific. Microplastics were more greatly correlated with proventricular versus cloacal microbiomes. Moreover, an increase in microplastics was associated with a decrease in commensal microbiota and an increase in (zoonotic) pathogens and antibiotic-resistant and plastic-degrading microbes. Though we note that these results were obtained from 16S rRNA gene sequencing, which has limitations in terms of reaching taxonomic identity at the strain level and identifying microbial functions, our study supports previous predictions that chronic microplastic ingestion is associated with gut dysbiosis^2^.

Our study illustrates that environmentally relevant microplastic concentrations and mixtures correlate with gut microbial diversity, highlighting the potential for gut dysbiosis in remotely living wild seabirds with large-scale migration routes and known to ingest microplastics debris in the wild^12–14^. Northern fulmars are well-known bioindicators of microplastic pollution^45^. Our results provide the basis upon which future research can examine the cumulative negative impacts of microplastics due to chronic exposure, especially considering that microplastics can be retained for weeks or months in Procellariiformes and thus unlikely to depend entirely on the last meal^46^. The implications are far-reaching: for one, humans are also exposed to micro- (and nano-) plastics^47–49^, raising the question of how humans and their (gut) health might be affected by plastic ingestion. For another, the gut microbiome plays a central role in host health, and as zoonoses and the state of wildlife health in a globalized world gain more attention^50^, the search for causes and origins of possible future zoonoses is gaining importance.

## Methods

### Sample collection

This study was conducted within the framework of two ongoing projects that, respectively, monitor Cory’s shearwaters (*C. borealis*) at the edge of the North Atlantic subtropical gyre on the Azores archipelago (Portugal) and northern fulmars (*F. glacialis*) near Qikiqtarjuaq, Nunavut in the Northwest Atlantic (Extended Data Fig. 1; BirdLife International^51^). Both species belong to the Procellariidae family, are surface-feeders and ingest microplastic debris^12–14,52,53^. Collections of northern fulmars were done during the breeding season between July and August 2018. A total of 27 northern fulmar adults were shot away from breeding sites^13^. Collections of Cory’s shearwaters were done during the take-off season when fledglings are known to collide with buildings and other manmade structures when abandoning the nest, often due to sensitivity towards artificial night light pollution, which can lead to death. Fresh fledgling corpses were then collected near colonies on the Azores between October and November 2017 and 2018. Birds were frozen at −20 °C until time of dissection and microbiome sampling, leading to a total of 58 collected fledgling individuals.

The collected seabirds were dissected and sampled under sterile conditions following a standardized protocol^15,54^. In addition to this protocol, sterile swabs were used to sample the proventriculus and cloaca of each individual bird (with the exception of one northern fulmar individual, which was mistakenly sampled only once at the proventriculus). Each swab was placed in nucleic acid preservation buffer^55^ and kept at −20 °C until DNA extraction. Plastic debris collected from the GIT over a 1 mm sieve was collected, examined under a light microscope and characterized following the protocols outlined in ref. ^56^. For brevity, we refer to this plastic debris as microplastic, even though not every piece measured was smaller than 5 mm (Rodríguez et al., in preparation)^13^. Thus, for each of the 85 seabird individuals, we collected data on the body mass and tarsus length of the individual, the number and total mass of microplastics in its GIT (using a balance with an accuracy of ±0.0001 g), and its proventricular (*n* = 85) and cloacal (*n* = 84) microbiome.

### DNA extraction, amplification and sequencing

DNA from whole swabs was extracted using Macherey-Nagel’s NucleoSpin ‘DNA from soil’ extraction kit (Germany) following the manufacturer’s protocol. An additional bead-beating step was incorporated into the protocol to mechanically lyse bacterial cells^6^. Throughout this step, 16 extraction blanks containing only the extraction reagents were included and subsequently sequenced to be able to identify and remove possible contamination.

Following DNA extraction, we targeted the V4 hypervariable region of the 16S rRNA gene using the bacterial primers 515F (5′-GTGCCAGCMGCCGCGGTAA-3′) and 806R (5′-GGACTACHVGGGTWTCTAAT-3′) (ref. ^57^), to which we added forward-primer (CS1-515F) and reverse-primer adapters (CS2-806R) to be able to use Fluidigm sequencing chemistry (Access Array System for Illumina Sequencing Systems, Fluidigm Corporation). We amplified this target region in two polymerase reaction chain steps (two-step PCR; Supplementary Information), ensuring to include PCR blanks comprising only the PCR reagents throughout this process. In the first step, a total PCR volume of 10 µl composed of 1 µl extracted DNA (5–10 ng), 1.5 µl (200 nM) pooled forward and reverse primers, 5 μl AmpliTaq Gold 360 Master Mix and 2.5 µl ultrapure dH_2_O was run under the following PCR conditions: initial denaturation at 95 °C for 10 min; 40 cycles including denaturation at 95 °C for 30 s, annealing at 60 °C for 30 s, and elongation at 72 °C for 45 s; final elongation at 72 °C for 7 min. Gel electrophoresis for each sample was conducted to ensure PCR success. The second PCR step consisted of a 20 µl PCR volume that included 2 µl amplified DNA from the first PCR step, 4 μl (400 nM) pooled forward and reverse barcode primers, 10 μl AmpliTaq Gold 360 Master and 4 µl ultrapure dH_2_O, which was run under the same PCR conditions as previously described in ten cycles. Barcoded samples were bead-purified (1:1 ratio), quantified and pooled to 8 nM. We paired-end sequenced a total of 169 swab samples, 16 extraction blanks and 5 PCR blanks in one run using our own in-house Illumina MiSeq sequencing platform at the Institute of Evolutionary Ecology and Conservation Genomics, Ulm University, Germany.

### Bioinformatic processing

Reads resulting from the Illumina MiSeq amplicon sequencing were processed in QIIME 2 (version 2020.8.0) using the DADA2 plug-in to generate ASVs^58,59^. Taxonomy was assigned with the QIIME 2 classify-sklearn function (and its default confidence value settings) using the SILVA (version 138) classifier trained using our target primers^60^. ASVs unassigned to bacteria at the domain level along with those identified as chloroplast or mitochondrial sequences were removed. We built a rooted phylogenetic tree as described in ref. ^6^. The phylogenetic tree, taxonomy and ASV tables along with sample metadata were imported into R (version 3.6.1) (ref. ^61^) to create a phlyoseq object using the phyloseq package (version 1.28.0) (ref. ^62^) for subsequent analyses.

In R, we first explored the extraction and PCR blanks that contained 185 out of a total of 2,956 ASVs. Of these 185 ASVs, 93 were unique to the blanks and subsequently removed. Using the decontam package (version 1.4.0) (ref. ^63^) with its prevalence-based contaminant identification and default threshold of 0.1, 18 additional ASVs were identified as possible contaminants and removed. We then considered samples with a sequencing depth of less than 2,900 reads as having failed and removed them and any ASVs unique to them from the dataset. Moreover, we applied a prevalence filter of 2% and an abundance filter of ten reads across the whole dataset to remove very rare ASVs that are likely to be sequencing artefacts. This removed 254 ASVs from the dataset and deleted all ASVs from extraction and PCR blanks. Following filtering, our dataset consisted of 4,602,578 reads across 2,517 ASVs and 169 samples, resulting in an average sequencing depth of 27,234 ± 5,999 reads per sample.

### Statistical analyses

To better reflect the effects microplastics may have on each individual, we expressed microplastic count and mass as proportions of each individual bird’s mass by dividing each individual’s microplastic count and mass by its body mass, though we also present results from using only the microplastic count or mass, without standardizing by bird mass. We used these variables throughout the analysis, though for brevity we refer to them as microplastic count and microplastic mass.

#### Alpha diversity

We first calculated intra-individual microbial diversity (alpha diversity) using the following metrics and packages: Faith’s PD, which reflects PD, (btools package, version 0.0.1) (ref. ^64^); Shannon index (log_e_), which takes both microbial richness and evenness into account; the observed number of ASVs, which reflects richness (the latter two both using the phyloseq package); and Allen’s H metric^65,66^. Then, using linear mixed effects models from the nlme package (version 3.1.141) (ref. ^67^), we modelled each alpha diversity metric as a function of the following: the interaction between microplastic count (scaled) and GIT location (either proventriculus or cloaca); the interaction between microplastic mass (scaled) and GIT location; host seabird species; and sequencing depth (scaled). We initially included the interaction between microplastic counts (scaled) and host seabird species plus the interaction between microplastic mass (scaled) and host seabird species to test if any microplastics effects on the gut microbiome are host species specific. However, not only did models with these two interactions have a worse fit than those without the interactions (using the Akaike information criterion (AIC), ΔAIC >2), neither interaction was statistically significant (*P* < 0.05), regardless of alpha diversity metric. Thus, we dropped these two interactions from our final models, kept host bird species alone as an explanatory factor, and concluded that any effect of microplastics on gut microbial alpha diversity was similar between fulmars and shearwaters, and not specific to either species. Moreover, we accounted for non-independence due to repeated sampling of the same individual at different points in the GIT (proventriculus and cloaca) by setting individual bird ID as a random factor (random intercept). The best model fit was obtained by square root transforming the observed number of ASVs and Allen’s H metric^68^. The remaining two alpha diversity metrics were not transformed. We accounted for different variances in alpha diversity between proventricular and cloacal microbiome samples, along with differences in variance according to sequencing depth by adding a varComb variance structure to the models, following the protocol outlined in ref. ^68^. We checked for multicollinearity between the explanatory variables using variance inflation factors from the car package (version 3.0.3) (ref. ^69^), which did not reveal any problematic variables^70^. Marginal (*R*^2^_LMM(m)_) and conditional (*R*^2^_LMM(c)_) *R*^2^ values^71^ for each model were calculated using the piecewiseSEM package (version 2.1.0) (ref. ^72^).

#### Beta diversity

To analyse the GIT microbial community composition in both seabird species, we generated distance matrices using the function phyloseq::distance based on weighted and unweighted UniFrac distances, since these were developed specifically for microbiome data^73^. In addition, we applied Aitchison’s log-ratio approach for compositional data^74^, which consists of centre-log transforming the ASV table after adding a pseudo count of one and generating a distance matrix using Euclidean distances. We then tested for effects of microplastics on microbial composition using null hypothesis testing with the permutation test implemented in the function vegan::adonis (version 2.5-5) (ref. ^23^). We defined one model per distance matrix (weighted and unweighted UniFrac, Aitchison) and used the same model formula as described in the previous section, with individual bird ID set within the ‘strata’ argument. To visualize the results of the multidimensional data, we used unconstrained ordination techniques PCoA for weighted and unweighted UniFrac matrices and principal component analysis for Aitchison’s approach using the function phyloseq::ordinate. To visually represent the effects of our discrete and continuous microplastics variables (count and mass), we fit these as vectors onto our ordination plots using the function vegan::envfit function based on the first two ordination axes.

#### Differential abundance analysis

To determine which microbial taxa could be driving the microplastics-associated differences in beta diversity, we performed an analysis of composition of microbiomes (ANCOM) test^25^. By adding linear mixed effects model functionality from the nlme package, we used the same model formula as previously described to determine which ASVs were associated with microplastic count and mass, with the interaction between microplastics and GIT location, and with the interaction between microplastics and host seabird species. Because zero inflation is a hallmark of microbiome data that can lead to false discovery rates in these types of analyses^25^, we applied an additional filter to keep only ASVs that were present in at least 15 samples. This reduced the total number of ASVs to 81. We then ran ANCOM using a significance level of 0.05, selected a moderate correction parameter to apply the Benjamini–Hochberg procedure that corrects for multiple testing^25^, and used the default cut-off value *w*_0_ = 0.70 so that only ASVs for which the null hypothesis was rejected at a rate of 70% or more were determined to be differentially abundant. To plot the differentially abundant ASVs and show which ASVs were positively or negatively correlated with microplastics, we adapted code from the QIIME 2 plug-in q2-composition^59^ for compositional data analysis to run in R and calculated model parameter estimates from the linear mixed model run on the ratio of each ASV-pair in the centred log-ratio transformed (clr) ASV table^25^.

#### Host body condition

To explore links between host body condition, microplastic ingestion and host gut microbiomes, we calculated body condition per seabird using the scaled mass index with individual tarsus length as a linear body measurement^75^. Then, we used a generalized linear model with a gamma log distribution and body condition as a response variable explained by microplastic count and mass, along with their interactions with sex and host species. Next, we included host body condition as an explanatory variable in our alpha and beta diversity models described above.

### Reporting summary

Further information on research design is available in the Nature Portfolio Reporting Summary linked to this article.

## Supplementary information

Supplementary Information Supplementary results.

Reporting Summary

Supplementary Tables 1–8 Legends for each table included in each Excel spreadsheet.

## Data Availability

Sequencing data and corresponding metadata are available on the National Center for Biotechnology Information under the accession number PRJNA930758. Additionally, the metadata are also stored on GitHub (https://github.com/gfackelmann/Current-levels-of-microplastic-pollution-impact-wild-seabird-gut-microbiomes).

## Code availability

The scripts for our analysis are stored on GitHub (https://github.com/gfackelmann/Current-levels-of-microplastic-pollution-impact-wild-seabird-gut-microbiomes).
