# Supplementary material for: Current levels of microplastic pollution impact wild seabird gut microbiomes
Source: Nat Ecol Evol. 2023 Mar 27;7(5):698–706. doi: 10.1038/s41559-023-02013-z (PMC10172112; doi:10.1038/s41559-023-02013-z)
Supplement: Supplementary file 1 — Supplementary results. [file 41559_2023_2013_MOESM1_ESM.pdf]

nature ecology & evolution

Article

https://doi.org/10.1038/s41559-023-02013-z

Current levels of microplastic pollution impact wild seabird gut microbiomes

In the format provided by the authors and unedited

Supplementary Information

Supplementary Results

Supplementary Results

Quantification of microplastics

The majority of individuals sampled (90.6%) contained microplastics in their gastrointestinal tract. The number of microplastics recovered ranged from zero to 57 pieces, with a median of 6 (IQR = 10). The mass of microplastics measured ranged from zero to 0.1016 g, with a median of 0.0145 g (IQR = 0.0297 g).

Impact of microplastics on microbial alpha diversity depends on location in the GIT but not host species

The model fit for our alpha diversity models was: $R^2_{LMM(m)} = 0.34$ and $R^2_{LMM(c)} = 0.34$ for observed numbers of ASVs; $R^2_{LMM(m)} = 0.26$ and $R^2_{LMM(c)} = 0.26$ for Shannon index; and $R^2_{LMM(m)} = 0.44$; $R^2_{LMM(c)} = 0.51$ for Faith's PD; and $R^2_{LMM(m)} = 0.39$ and $R^2_{LMM(c)} = 0.39$ for Allen's H metric. Aside from microplastics variables, we found significant correlations between all four alpha diversity metrics and GIT location (observed number of ASVs: $\beta = -1.61$, $t_{80} = -6.72$, $p < 0.001$; Shannon index: $\beta = -0.45$, $t_{80} = -4.48$, $p < 0.001$; Faith's PD: $\beta = -4.35$, $t_{80} = -8.67$, $p < 0.001$; Allen's H metric: $\beta = -0.17$, $t_{80} = -6.33$, $p < 0.001$), as well as host seabird species (observed number of ASVs: $\beta = 0.67$, $t_{81} = 2.26$, $p = 0.027$; Shannon index: $\beta = 0.35$, $t_{81} = 2.84$, $p = 0.006$; Faith's PD: $\beta = 1.72$, $t_{81} = 2.40$, $p = 0.019$; Allen's H metric: $\beta = 0.08$, $t_{81} = 2.55$, $p = 0.013$), whereas sequencing depth was only significantly correlated with Shannon index ($\beta = -0.15$, $t_{80} = -3.50$, $p < 0.001$) and Allen's H metric ($\beta = -0.03$, $t_{80} = -2.66$, $p = 0.009$; Supplementary Table 1).

Impact of microplastics on microbial beta diversity depends on location in the GIT and host species

In our permutation models, we also accounted for relevant non-microplastics variables that we listed first in these models, since order of variables plays a role in the vegan::adonis function that evaluates if the next variable explains significantly more variation than the previous one. Thus, we accounted for and found significant correlations between beta diversity and host species identity (weighted UniFrac $p < 0.001$; unweighted UniFrac: $p < 0.001$; Aitchison: $p < 0.001$), location of the microbiome within the GIT (weighted UniFrac $p < 0.001$; unweighted UniFrac: $p < 0.001$; Aitchison: $p < 0.001$), and host sex (weighted UniFrac $p < 0.001$; unweighted UniFrac: $p < 0.001$; Aitchison: $p < 0.001$), but not with sequencing depth (Supplementary Table 3).

Microplastics shift microbial taxonomic abundances in different parts of the GIT and in different host species

Using ANCOM, we found 17 ASVs to be differentially abundant according to microplastic count and mass. These 17 ASVs spanned four phyla (Actinobacteriota, Firmicutes, Fusobacteriota, and Proteobacteria), five classes (Actinobacteria, Bacilli, Clostridia, Fusobacteriia, and Gammaproteobacteria), 10 orders (Corynebacteriales, Actinomycetales, Lactobacillales, Staphylococcales, Clostridiales, Peptostreptococcales-Tissierellales, Fusobacteriales, Enterobacterales, Alteromonadales, and Pseudomonadales), and 12 families (*Corynebacteriaceae*, *Actinomycetaceae*, *Lactobacillaceae*, *Enterococcaceae*, *Catellicoccaceae*, *Staphylococcaceae*, *Clostridiaceae*, Peptostreptococcales-Tissierellales, *Fusobacteriaceae*, *Hafniaceae*, *Pseudoalteromonadaceae*, and *Moraxellaceae*).
