# Supplementary material for: Current levels of microplastic pollution impact wild seabird gut microbiomes
Source: Nat Ecol Evol. 2023 Mar 27;7(5):698–706. doi: 10.1038/s41559-023-02013-z (PMC10172112; doi:10.1038/s41559-023-02013-z)
Supplement: Supplementary file 2 — Reporting Summary [file 41559_2023_2013_MOESM2_ESM.pdf]

nature portfolio

Corresponding author(s): Gloria Fackelmann
Simone Sommer

Last updated by author(s): Feb 3, 2023

Reporting Summary

Nature Portfolio wishes to improve the reproducibility of the work that we publish. This form provides structure for consistency and transparency in reporting. For further information on Nature Portfolio policies, see our Editorial Policies and the Editorial Policy Checklist.

Statistics

For all statistical analyses, confirm that the following items are present in the figure legend, table legend, main text, or Methods section.

n/a	Confirmed	
☐	☒	The exact sample size (n) for each experimental group/condition, given as a discrete number and unit of measurement
☐	☒	A statement on whether measurements were taken from distinct samples or whether the same sample was measured repeatedly
☐	☒	The statistical test(s) used AND whether they are one- or two-sided<br>*Only common tests should be described solely by name; describe more complex techniques in the Methods section.*
☐	☒	A description of all covariates tested
☐	☒	A description of any assumptions or corrections, such as tests of normality and adjustment for multiple comparisons
☐	☒	A full description of the statistical parameters including central tendency (e.g. means) or other basic estimates (e.g. regression coefficient) AND variation (e.g. standard deviation) or associated estimates of uncertainty (e.g. confidence intervals)
☐	☒	For null hypothesis testing, the test statistic (e.g. F, t, r) with confidence intervals, effect sizes, degrees of freedom and P value noted<br>*Give P values as exact values whenever suitable.*
☒	☐	For Bayesian analysis, information on the choice of priors and Markov chain Monte Carlo settings
☒	☐	For hierarchical and complex designs, identification of the appropriate level for tests and full reporting of outcomes
☐	☒	Estimates of effect sizes (e.g. Cohen's d, Pearson's r), indicating how they were calculated

Our web collection on statistics for biologists contains articles on many of the points above.

Software and code

Policy information about availability of computer code

Data collection	MS Excel
Data analysis	QIIME 2 (version 2020.8.0), R (version 3.6.1), Dendroscope (version 3.5.10).

For manuscripts utilizing custom algorithms or software that are central to the research but not yet described in published literature, software must be made available to editors and reviewers. We strongly encourage code deposition in a community repository (e.g. GitHub). See the Nature Portfolio guidelines for submitting code & software for further information.

Data

Policy information about availability of data

All manuscripts must include a data availability statement. This statement should provide the following information, where applicable:

- Accession codes, unique identifiers, or web links for publicly available datasets
- A description of any restrictions on data availability
- For clinical datasets or third party data, please ensure that the statement adheres to our policy

Sequencing data are available on NCBI under the accession number PRJNA930758.

Field-specific reporting

Please select the one below that is the best fit for your research. If you are not sure, read the appropriate sections before making your selection.

☐ Life sciences ☐ Behavioural & social sciences ☒ Ecological, evolutionary & environmental sciences

For a reference copy of the document with all sections, see nature.com/documents/nr-reporting-summary-flat.pdf

Ecological, evolutionary & environmental sciences study design

All studies must disclose on these points even when the disclosure is negative.

Study description	We investigated if accumulated microplastics are associated with changes in the proventricular and cloacal microbiomes in two seabird species that chronically ingest microplastics, northern fulmars (Fulmarus glacialis, n=27 individuals) and Cory's shearwaters (Calonectris borealis, n=58 individuals). Each bird was swabbed twice (with one exception) at the proventriculus (n = 85 microbiome proventricular samples) and cloacal (n = 84 cloacal microbiome samples). We modeled the interaction between microplastic count (scaled) and GIT location (either proventriculus or cloaca); the interaction between microplastic mass (scaled) and GIT location; the interaction between microplastic counts (scaled) and species plus the interaction between microplastic mass (scaled) and host bird species to test if any microplastics effects on the gut microbiome are GIT location or host species-specific.
Research sample	One population of northern fulmars (Fulmarus glacialis) collected near Qikiqtarjuaq, Nunavut, Canada and one population of Cory's shearwaters (Calonectris borealis) collected at the edge of the North Atlantic subtropical gyre on the Azores archipelago (Portugal), both with samples from both sexes. Northern fulmars were breeding adults and Cory's shearwaters were fledglings. These species were chosen because they are known to ingest microplatics. Moreover, the northern fulmar is a bioindicator of environmental microplastic pollution.
Sampling strategy	Collections of Cory's shearwaters were done during the take-off season when fledglings are known to collide with buildings and other manmade structures when abandoning the nest, often due to sensitivity towards artificial night light pollution, which can lead to death. Collections of northern fulmars were done during the breeding season, when they are more easily accessible for collection. Microbiome sampling was integrated into these two existing seabird monitoring and sampling projects to minimize the need for additional loss of life.
Data collection	Microbial swabs were collected during laboratory necroposies of the seabird individuals conducted by Yasmina Rodríguez, Jennifer F. Provencher, and Julia E. Baak.
Timing and spatial scale	Collections of northern fulmars were done during the breeding season between July and August 2018. Collections of Cory's shearwaters were done during the fledgling take-off season between October and November 2017 and 2018. Collected dates were determined by seabird breeding and take-off seasons and in order to maximize sample sizes.
Data exclusions	In R, we first explored the extraction and PCR blanks that contained 185 out of a total of 2,956 ASVs. Of these 185 ASVs, 93 were unique to the blanks and subsequently removed. Using the decontam package (version 1.4.0) with its prevalence-based contaminant identification and default threshold of 0.1, 18 additional ASVs were identified as possible contaminants and removed. We then considered samples with a sequencing depth of less than 2,900 reads as having failed and removed them and any ASVs unique to them from the dataset. Moreover, we applied a prevalence filter of 2% and an abundance filter of 10 reads across the whole dataset to remove very rare ASVs that are likely to be sequencing artifacts. This removed 254 ASVs from the dataset and deleted all ASVs from extraction and PCR blanks. Following filtering, our dataset consisted of 4,602,578 reads across 2,517 taxa and 169 samples, resulting in an average sequencing depth of 27,234 ± 5,999 reads per sample.
Reproducibility	All attempts to repeat the experiment were successful.
Randomization	Swab samples were extracted in a random order.
Blinding	Authors who collected swab samples did not conduct the microbiome analysis.

Did the study involve field work? ☒ Yes ☐ No

Field work, collection and transport

Field conditions	Seabirds were collected near Qikiqtarjuaq, Nunavut, Canada between July and August 2018 and at the edge of the North Atlantic subtropical gyre on the Azores archipelago (Portugal) between July and August 2018.
Location	Sampling was conducted near Qikiqtarjuaq, Nunavut, Canada and at the edge of the North Atlantic subtropical gyre on the Azores archipelago (Portugal).
Access & import/export	Northern fulmars were collected with animal care permits (Acadia University Animal Care Committee Permit 02-18), federal permits for work on seabirds and in National Wildlife Areas (ECCC NUN-NWA-18-02, NUN-SCI-18-02), and territorial permits (GN-WL-2018-004; NIRB-17YN069; NPC-148645). The dead corpses of shearwaters were collected within the framework the SOS Cagarro Campaign organized by the Direção Regional dos Assuntos do Mar (DRAM), with help from the Direção Regional do Ambiente (DRA) and the Parques Naturais de Ilha (PNI).

Disturbance	Northern fulmars were collected while feeding away from breeding colonies in collaboration with local Inuit hunters near Qikiqtarjuaq, Nunavut. Cory's shearwaters were already dead upon collection.

Reporting for specific materials, systems and methods

We require information from authors about some types of materials, experimental systems and methods used in many studies. Here, indicate whether each material, system or method listed is relevant to your study. If you are not sure if a list item applies to your research, read the appropriate section before selecting a response.

Materials & experimental systems

n/a	Involved in the study	
☒	☐	Antibodies
☒	☐	Eukaryotic cell lines
☒	☐	Palaeontology and archaeology
☐	☒	Animals and other organisms
☒	☐	Human research participants
☒	☐	Clinical data
☒	☐	Dual use research of concern

Methods

n/a	Involved in the study	
☒	☐	ChIP-seq
☒	☐	Flow cytometry
☒	☐	MRI-based neuroimaging

Animals and other organisms

Policy information about studies involving animals; ARRIVE guidelines recommended for reporting animal research

Laboratory animals	This study did not involve laboratory animals.
Wild animals	Cory's shearwaters were already dead upon collection. Northern fulmars were shot using 12-gauge shotguns while feeding away from breeding colonies in collaboration with local Inuit hunters near Qikiqtarjuaq, Nunavut. Carcasses were transported in coolers until they could be stored in freezers. Both species had individuals from both sexes. Northern fulmars were breeding adults and Cory's shearwaters were fledglings.
Field-collected samples	Seabird carcasses were transported in coolers until they could be stored in freezers.
Ethics oversight	Northern fulmars were collected with animal care permits (Acadia University Animal Care Committee Permit 02-18), federal permits for work on seabirds and in National Wildlife Areas (ECCC NUN-NWA-18-02, NUN-SCI-18-02), and territorial permits (GN-WL-2018-004; NIRB-17YN069; NPC-148645). The dead corpses of shearwaters were collected within the framework the SOS Cagarro Campaign organized by the Direção Regional dos Assuntos do Mar (DRAM), with help from the Direção Regional do Ambiente (DRA) and the Parques Naturais de Ilha (PNI). Okeanos received national funds through the FCT – Foundation for Science and Technology, I.P., under the project UIDB/05634/2020 and UIDP/05634/2020 and through the Regional Government of the Azores (M1.1.A/REEQ.CIENTÍFICO UI&D/2021/010). JFP and MLM were supported by the Northern Contaminants Program (Crown-Indigenous Relations and Northern Affairs Canada).

Note that full information on the approval of the study protocol must also be provided in the manuscript.
